# 3D quasi-skyrmions in thick cylindrical and dome-shape soft nanodots

**DOI:** 10.1038/s41598-022-07407-w

**Published:** 2022-03-02

**Authors:** Eider Berganza, Jose Angel Fernandez-Roldan, Miriam Jaafar, Agustina Asenjo, Konstantin Guslienko, Oksana Chubykalo-Fesenko

**Affiliations:** 1 Institute of Nanotechnology, KIT, 76344 Eggenstein-Leopoldshafen, Germany; 2 Instituto de Ciencia de Materiales de Madrid, CSIC, 28049 Madrid, Spain; 3 Department of Physics, University of Oviedo, 33007 Oviedo, Spain; 4 Departamento de Física de la Materia Condensada and Condensed Matter Physics Center (IFIMAC), Universidad Autónoma de Madrid, 28049 Madrid, Spain; 5 Departamento de Polímeros y Materiales Avanzados, University of the Basque Country (UPV/EHU), 20018 Donostia, Spain; 6 IKERBASQUE, The Basque Foundation for Science, 48009 Bilbao, Spain

**Keywords:** Magnetic properties and materials, Ferromagnetism

## Abstract

Magnetic skyrmions are widely attracting researchers due to fascinating physics and novel applications related to their non-trivial topology. Néel skyrmions have been extensively investigated in magnetic systems with Dzyaloshinskii–Moriya interaction (DMI) and/or perpendicular magnetic anisotropy. Here, by means of micromagnetic simulations and analytical calculations, we show that 3D quasi-skyrmions of Néel type, with topological charge close to 1, can exist as metastable states in soft magnetic nanostructures with no DMI, such as in Permalloy thick cylindrical and dome-shaped nanodots. The key factor responsible for the stabilization of DMI-free is the interplay of the exchange and magnetostatic energies in the nanodots. The range of geometrical parameters where the skyrmions are found is wider in magnetic dome-shape nanodots than in their cylindrical counterparts. Our results open the door for a new research line related to the nucleation and stabilization of magnetic skyrmions in a broad class of nanostructured soft magnetic materials.

## Introduction

Magnetic skyrmions are topologically non-trivial magnetization configurations which typically appear in systems with strong Dzyaloshinskii-Moriya interactions (DMI) and broken inversion symmetry. Skyrmion lattices have been widely observed in crystals with intrinsic non-centro-symmetric lattices^1,2^ and individual skyrmions—in thin multilayered films composed of transition metals and high-spin orbit coupling materials such as Co/Pt, Ir/Co/Pt and similar^3,4^. The above multilayered thin films also have perpendicular magnetic anisotropy (PMA), with an out-of-plane easy axis. PMA together with DMI ensure either Bloch or Néel (or mixed) skyrmions stabilization, typically as metastable states at zero bias magnetic field^5,6^. Both multilayered films^7–9^ as well as B20 single crystals^10^ can be appealing to controllably nucleate and drive skyrmions using external stimuli. An additional requirement is a good stability of the individual skyrmions at room temperature. The search for new materials which ensure thermally stable and small radius skyrmions, involves engineering the competition between DMI and PMA^11^. However, both DMI and PMA are not absolutely necessary for skyrmion existence. Indeed, Bloch skyrmions (or classical bubbles) can be stabilized by magnetostatic interaction with no need of DMI^12,13^ in systems with PMA, or as a consequence of precessional dynamics in the case of dynamic skyrmions^14^. According to the standard definition, the magnetization profile of Bloch skyrmions have azimuthal magnetization component while Néel skyrmions possess only radial component. Néel skyrmions can be also stabilized in systems with in-plane anisotropy^15^ and DMI. However, so far, for the existence of chiral Néel skyrmions, DMI has been considered as a necessary ingredient.

When it comes to real applications, skyrmions may need to be confined in nanostructured geometries, such as for instance magnetic stripes or dots. Confinement is indeed an additional factor which favors the appearance of topologically non-trivial magnetic configurations due to the influence of the magnetostatic energy^16–18^. Complex 3D magnetic textures such Bloch have been reported in modelling in magnetically soft magnetic spheres^19–21^ and recently observed experimentally in asymmetric Permalloy disks^22^. The Bloch point is also a natural magnetization configuration for a domain wall center in cylindrical nanowires^23^. Several ingredients could favor stabilization of skyrmions in soft magnetic nanostructures with no DMI. First, in cylindrical nanopillars (with large aspect ratio height-to-diameter) the effect of the magnetostatic interactions leads to an effective out-of-plane anisotropy. Secondly, in ultra-thin magnetic caps with curved geometry, a part of the exchange integral on the curvilinear surface was shown to have functional form similar to DMI^24–26^. In this sense, the curved geometry itself may produce similar effects to that of DMI systems, with no need of real DMI of the relativistic origin and therefore leading to less constraints in the choice of materials. Thus, it is natural to expect existence of the skyrmions (both Bloch and Néel) in thick cylindrical dots. Their region of stability may be further improved by curved surfaces. Our group has recently reported the observation of half-hedgehog spin textures, nucleated and further stabilized by the MFM probe stray field^27^. Recent numerical simulations^26,27^ also demonstrated the existence of skyrmions in hemispherical nanoparticles with no DMI but with PMA. However, up to our knowledge, Néel skyrmions have not been reported in systems with no DMI nor any magnetic anisotropy.

Here we conduct micromagnetic simulations and analytical calculations of soft Permalloy magnetic dots with cylindrical or dome-shaped geometries with neither PMA nor DMI. The occurrence of the vortex state and Bloch points is widely reported in nanodisks and spheres^19–21,28^. Apart from these magnetization configurations, we obtained a 3D quasi-skyrmion, (a spin texture having a radial configuration and a topological charge close to one in the basal plane), as a metastable state in dome-shaped nanodots and we have compared it to that in thick cylindrical dots. The spherical geometry also leads to a coupling between the skyrmion polarization and the radial magnetization components. We present the state diagram for 3D quasi-skyrmions in soft magnetic dome-shape nanodots and nanopillars made of Permalloy in terms of the dot geometry (radius and height). Finally, we have also calculated 2D topological charges displaying strong dependence on the sample geometry in both types of the patterned nanostructures.

## Results

Micromagnetic simulations were conducted in 3D soft magnetic dots with cylindrical or curved (dome-shape) geometry with neither PMA nor DMI, nor external magnetic fields. The geometries of both magnetic elements correspond to nanodots of base radius in the range 10–60 nm. The aspect ratio (height/radius) was varied between 0.1 and 1. Further modelling details can be found in the “Methods” section.

### Magnetic dome-shape nanodots

Magnetic dome-shape nanodots are cut spheres that, similarly to spherical particles^19–21^, might host different 3D stable and metastable magnetization states, depending on the nucleation path. While searching for skyrmions in numerical simulations, we have found that in a wide range of parameters magnetic domes possess multiple metastable states, i.e., the resulting magnetization configuration depends on the initial conditions.

**Figure 1:**
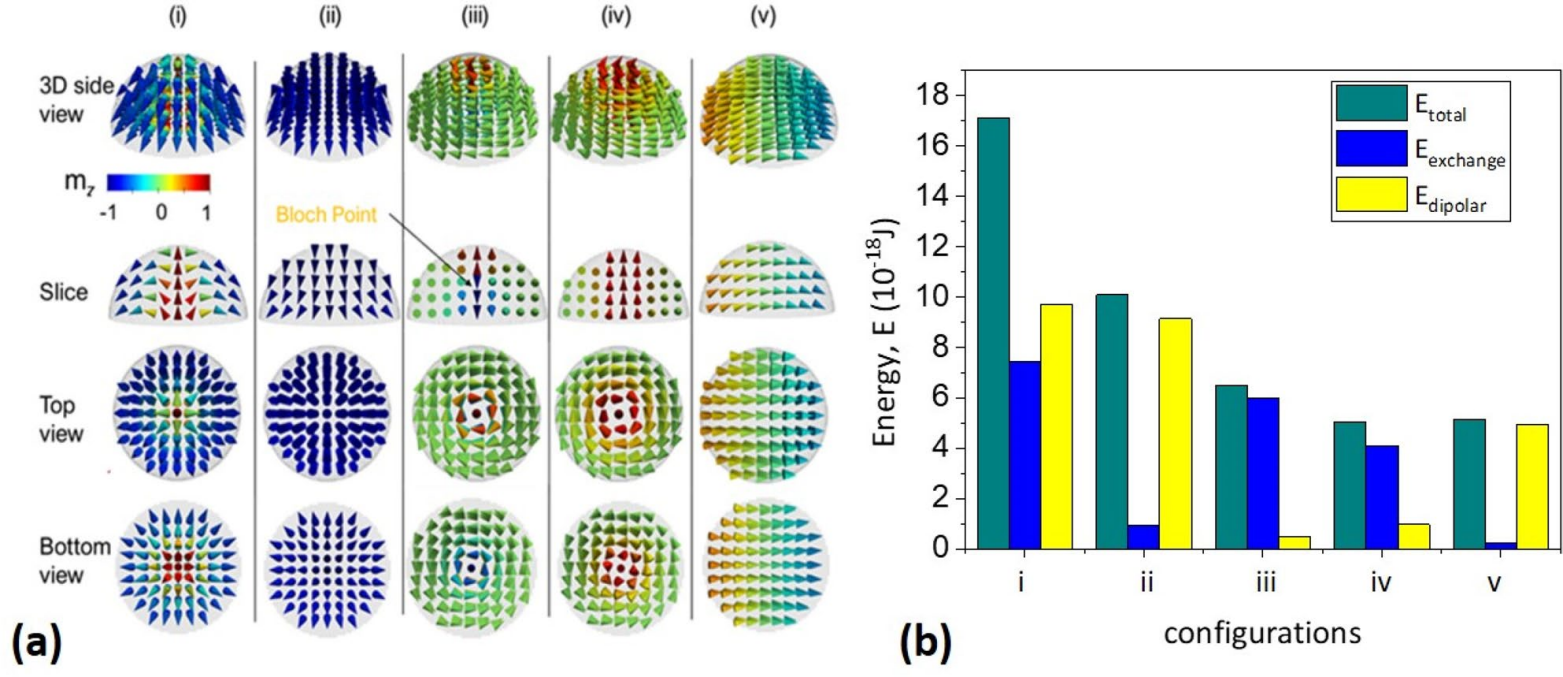
(**a**) Magnetic configurations (corresponding to the different magnetic energy minima) in Permalloy hemispherical nanodots of radius *R* = 30 nm: (**i**) 1—Néel quasi-skyrmion, (**ii**) 2—‘Flower-like’ state, (**iii**) 3—Bloch point, (**iv**) 4—vortex and (**v**) 5—in-plane single domain (SD). Arrows stand out for the direction of the magnetization and are coloured according to the out-of-plane magnetization component. (**b**) The different contributions and total magnetic energy values of these magnetization configurations.

Figure 1 displays results of micromagnetic modelling with different initial conditions, showing minimum energy magnetic states of a hemispherical Permalloy nanodot of 30 nm radius. Several spin-textures were obtained for this geometry: (1) 3D Néel-like quasi-skyrmion, having a configuration of the radial vortex in the basal plane, (2) ‘Flower-like’ (near to an out-of-plane configuration, with curling magnetization on the edges to minimize demagnetization energy) state^29^, (3) Bloch point in the middle of the hemisphere (with a singularity of the magnetization in the middle plane)^21^, (4) vortex and (5) in-plane quasi-single domain (SD) states. See also the videos in Sect. 1 of the “Supplementary Information” to visualize the three-dimensional arrangement of magnetic moments in the nanodot for each magnetic state displayed in Fig. 1a. The total magnetic energies of these states are compiled in Fig. 1b, together with the exchange and magnetostatic contributions. Overall, the vortex state corresponds in this case to the magnetization ground state and is characterized by the minimization of the magnetostatic energy. The in-plane single domain configuration presents an energy value slightly higher than that of the vortex state (5 × 10^–18^ J), in spite of the minimization of the exchange energy. The deviations of the magnetization from the uniform in-plane state are related to a minimization of the magnetostatic energy at the boundaries of soft isotropic non-ellipsoidal magnetic materials^30^. The Bloch point and the ‘flower-like’ state possess higher energies than the above-mentioned configurations. The energy of the Bloch point is slightly above the vortex configuration energy due to their similar configurations in the cross-sections, with the addition of a singularity in the particle center with an excess of the exchange energy. The ‘flower-like’ state, similar to the out-of-plane SD state, also shows deviations of the magnetization from the uniform state along the ‘hard direction’ of the configurational anisotropy and has a higher energy than the in-plane SD one. A 3D Néel-like skyrmion is the most energetic state, with its energy value being three times higher (1.71 × 10^-17^ J) than that of the vortex. This state is probably stabilized by the curvature of the surface.

**Figure 2:**
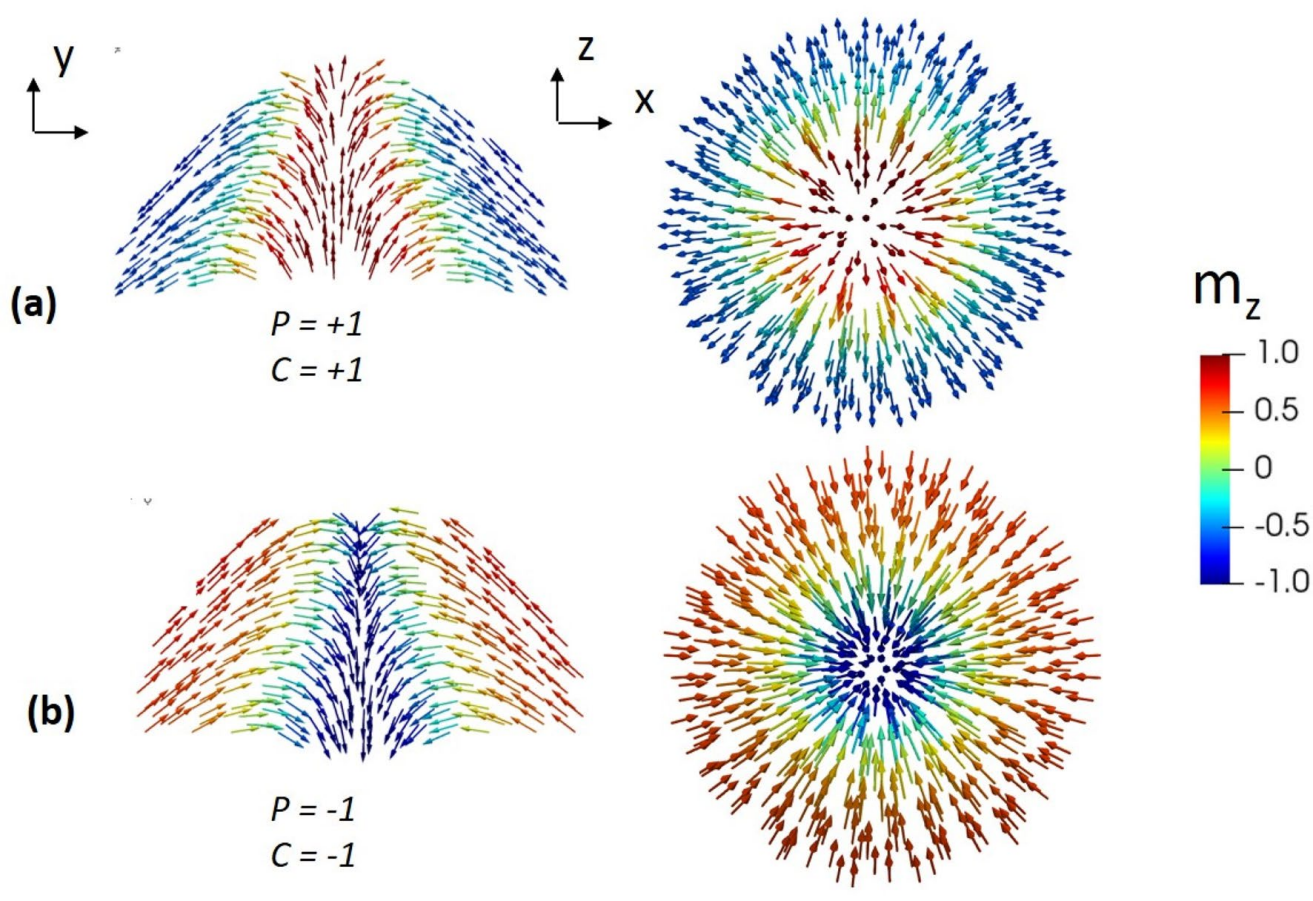
Two possible Néel skyrmion magnetization configurations obtained in a hemispherical dot with (**a**) positive core polarity (P) and outwards radial chirality (C) and (**b**) negative core polarity and inwards radial chirality. The vertical cross-section and basal plane configurations are shown. The dot radius is 30 nm.

Thus, the simulations proved that a 3D Néel-like skyrmion configuration is, at least, metastable in the Py hemispheres of 30 nm radius. A closer look at this spin texture shows that the magnetization configuration is three dimensional, and more complex than simple hedgehog configuration due to the sample curvature effect. Its core diameter is wider at the base of the nanodot and it narrows approaching the nanodot upper surface (Fig. 2). On the other hand, as it has been previously reported^27^, we observed that curved surfaces introduce a coupling between the polarity (magnetization direction of the core) and the radial magnetization components of the outer part. In the investigated soft magnetic domes, the signs of the radial magnetization component *m*_ρ_ (chirality C) and the skyrmion core polarity P = sign (*m*_z_ (r = 0)) are not independent and fulfill the condition P*C = 1 due to the influence of the magnetostatic energy, see Fig. 2. Indeed, the other two possible core polarity–chirality combinations have not been obtained in micromagnetic simulations under the same conditions. We have found that they can be stabilized by, for example, introducing additional radial^27^ or perpendicular^26^ magnetic anisotropy (see Sect. 2 of the “Supplementary Information”, where a magnetization configuration with P*C = − 1 is presented). Thus, in a completely magnetically soft case, Néel skyrmions in dome-shaped nanodots display chiral nature.

**Figure 3:**
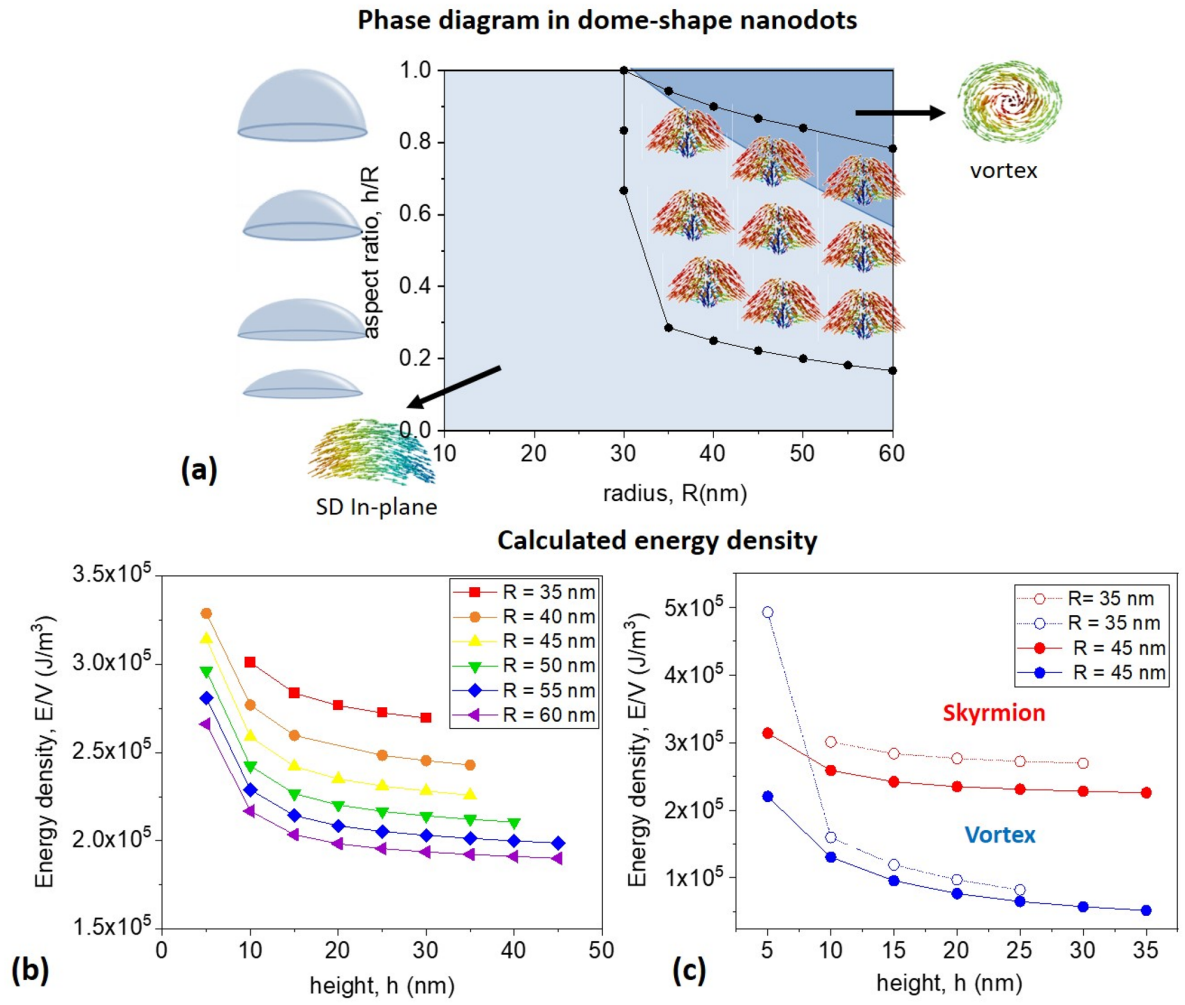
(**a**) Simulated phase diagram of a soft magnetic dome-shaped nanodot, the area where the Néel skyrmion exists is bound inside the black curves. The light blue colour indicates the region where the SD state is the ground state of the system while the blue—the same for the vortex state. Inset corresponds to the magnetisation configuration in a nanodot of radius *R* = 30 nm and height *h* = 30 nm. (**b**) Calculated energy density curves for dome-shaped nanodots of increasing radius values, as a function of dot height. (**c**) Comparison of the vortex, blue symbols and Néel skyrmion, red symbols, energy densities for the dome-shaped nanodots of 35 and 45 nm radii, as a function of the dot height.

Additionally, to unveil the geometrical parameters region where quasi-skyrmionic spin-textures exist, further simulations were conducted. Figure 3a presents the state diagram in terms of the dome radius *R* and height *h*, where Néel skyrmion-like configurations were obtained. The region of interest is delimited by the black dots and lines in Fig. 3a and skyrmions appear starting with the dot radius *R* ca. 30 nm. Skyrmions are always metastable states being either the SD state (light grey color in Fig. 3a) or the vortex state (dark grey color) the ground states. The skyrmion energy density decreases as the aspect ratio *h/R* increases (see Fig. 3b) and it decreases with the dot radius. Thus, it is difficult to stabilize skyrmions in dots of very small radius due to the increase of the exchange energy. We have found that the vortex state exists in a large interval of the geometrical parameters and in the whole region of the skyrmion existence. The vortex always has a smaller energy than that of the Néel skyrmion, see Fig. 3c.

In order to better understand the topologically non-trivial nature of our magnetization configuration. we have calculated the value of the 2D topological charges for different cross-sections across the nanodot height. Although, in the literature different approaches have been proposed to describe the topology of three-dimensional magnetization configurations, such as the calculation of the Hopf index^31–33^ or the integration of the gyrovector over a closed surface (which is quite relevant for the description of magnetization dynamics under the application of magnetic fields or currents^34^), its proper notion is still a subject of debate. In our case, we have chosen to represent the 2D topological charge across the dot height as this allows direct comparison^23^ with cylindrical thin nanodots. Thus, we have calculated the 2D topological charge values in different dot cross-sections as a function of out-of-plane (vertical) coordinate, *z*.

1 $$Q=\frac{1}{4\pi }\underset{S}{\overset{}{\int }}\mathbf{m}\cdot \left(\frac{\partial {\varvec{m}}}{\partial x}\times \frac{\partial {\varvec{m}}}{\partial y}\right)dxdy$$

**Figure 4:**
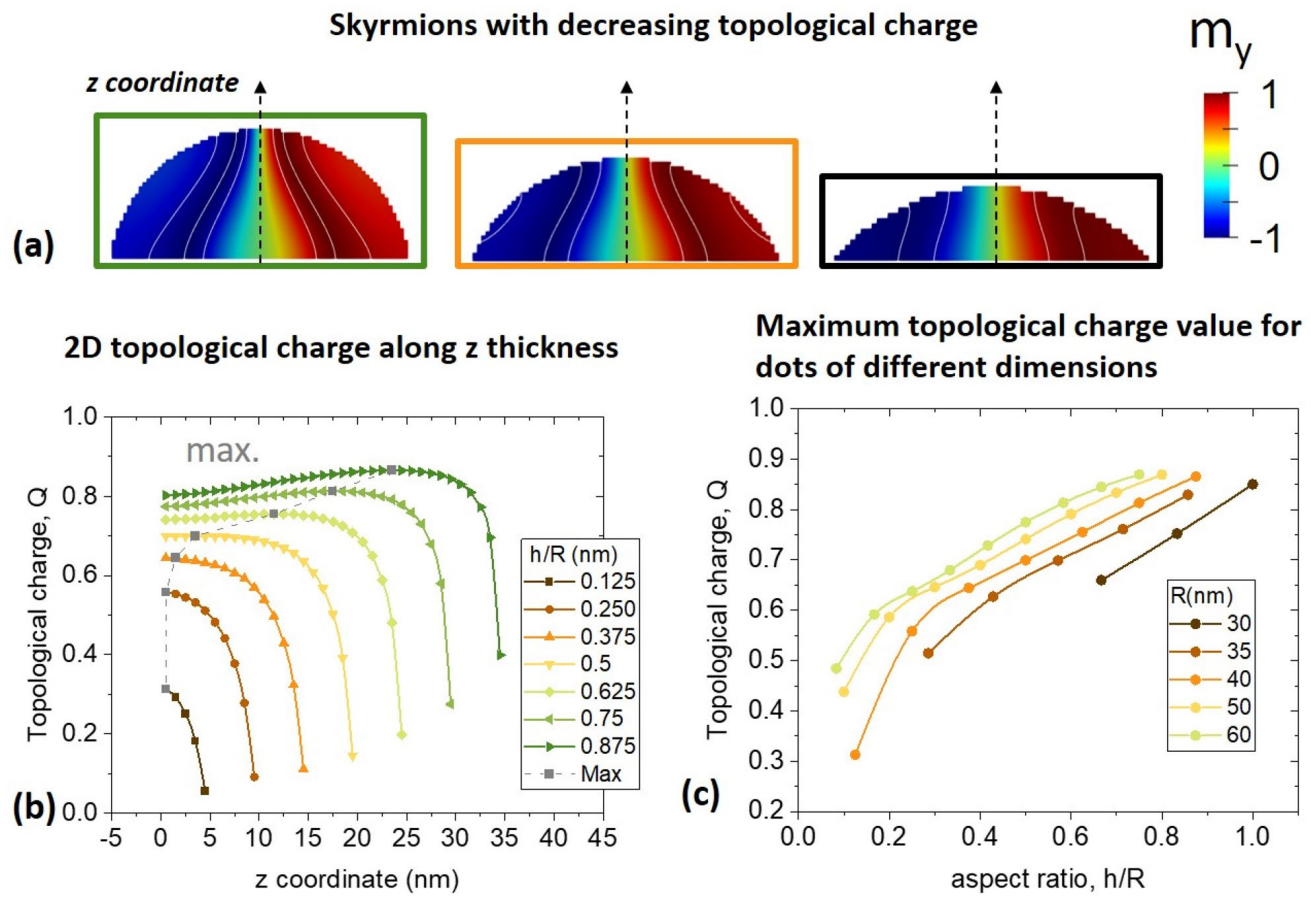
(**a**) Skyrmionic configurations in domed-shaped nanodots of radius 40 nm and aspect ratios *h/R* = 0.875, 0.375 and 0.125. (**b**) Calculated 2D topological charge for nanodots of radii 40 nm and increasing aspect ratios, as a function of the coordinate *z* along the direction perpendicular to the dot basal plane. The grey dash line joins points of maximum topological charge in each curve. (**c**) Maximum 2D topological charge of the dome-shaped dots of increasing radii values, as a function of their aspect ratio.

The results are presented in Fig. 4b, for a dome-shaped dot of *R* = 40 nm varying its height (Fig. 4a). Notice that the 2D topological charge of the 3D magnetization configuration changes as a function of the vertical coordinate, *z*. Furthermore, its maximum value shown in Fig. 4c, does not lay on the bottom, as its position is displaced to higher *z*-values. Dots with the larger aspect ratios, achieve a maximum value of the topological charge at a point around the middle of the structure height, while in dots with lower aspect ratios the maximum topological charge is achieved always at the bottom of the dot. Importantly, the topological charge values are smaller than 1 due to the influence of the sample boundary, while values close to it are observed for larger nanodot heights. As a result, as of now we refer to the studied magnetization configuration as 3D quasi-skyrmion. As shown in Sect. 3 of the “Supplementary Information”, the integration of the 2D topological charge given by Eq. (1) over *z*-coordinate yields the *z*-component of the global gyrovector of the 3D magnetization configuration. Direct computation shows that other components of the gyrovector are negligible.

The maximum value of the topological charge is presented in Fig. 4c for various radii for the cases when skyrmions exist. This value increases with the increase of the dot radius and its height, approaching the values close to one. Skyrmions in dome-shaped nanodots with small heights have small charges, even smaller than 0.5. Because of that, the lower boundary for the skyrmion existence was depicted with a dashed line in Fig. 3a, since it is therefore subject to the criteria used to define a skyrmion, in terms of its topological charge. The results also demonstrate the influence of the curvature, since given the same base radius, larger topological charges are obtained in thicker nanodots.

Coming back to other configurations described in Fig. 1, the 3D vortex state (Fig. 1a, iii) has a topological charge 0.5 at the base plane decreasing along the out-of-plane coordinate. Also, the quasi-out-of-plane state in Fig. 1a (ii) has a small topological charge due to the curvature effect. However, its value does not exceed 0.2 at the basal plane and decreases in the out-of-plane direction. These results are presented in Sect. 4 of “Supplementary Information” in details.

### Skyrmions in thick cylindrical nanodisks

The above results unambiguously demonstrate that 3D quasi-skyrmions can be stable in soft magnetic domes, i.e., in curved geometries. The next question we address is whether curvature is a necessary condition or, on the contrary, if the skyrmion can still exist in a thick cylindrical nanodot (nanopillar) and the curvature just adds stability to it. Since with numerical simulations many initial conditions are necessary for the search of metastable configurations, we have performed analytical analysis of both vortex and Néel skyrmion stability in cylindrical dots with similar dimensions.

To calculate the magnetic energy of the skyrmions in this geometry, we parameterize the unit magnetization vector by the spherical angles ${\varvec{m}}={\varvec{m}}\left(\Theta ,\Phi \right)$. The angles $\Theta ,\Phi$ are functions of the radius vector ${\varvec{r}}=\left(\rho ,\varphi ,z\right)$ represented by the cylindrical coordinates. The total magnetic energy functional is $E\left[{\varvec{m}}\right]=\int dV\varepsilon \left({\varvec{m}}\right)$, with the energy density $\varepsilon \left({\varvec{m}}\right)=A{\left(\nabla {\varvec{m}}\right)}^{2}+{\varepsilon }_{m}\left({\varvec{m}}\right)$, where *A* is the exchange stiffness constant, and ${\varepsilon }_{m}$ is the magnetostatic energy density. We assume that a skyrmion equilibrium configuration does not depend on the height coordinate *z* and is radially symmetric, i.e., $\Theta =\Theta \left(\rho \right)$, $\Phi =\varphi +{\varphi }_{0}$ (the helicity ${\varphi }_{0}=0,\uppi$ for the Néel skyrmions and ${\varphi }_{0}=\pm \pi /2$ for the vortices or Bloch skyrmions). Then, the total magnetic energy as a functional of the skyrmion magnetization is solely represented by the polar angle $\Theta \left(\rho \right)$, $E=E\left[\Theta \left(\rho \right)\right]$. The energy in units of *µ*_*0*_*M*_*s*_^2^*V* (*V* is the dot volume) is

2 $$e\left[\Theta \left(r\right)\right]=\frac{{l}_{ex}^{2}}{{R}^{2}}{\int }_{0}^{1}drr\left[{\left({\Theta }_{r}^{^{\prime}}\right)}^{2}+\frac{1}{{r}^{2}}{\mathit{sin}}^{2}\Theta \right]+{e}_{m}\left[\Theta \left(r\right)\right]$$

where $r=\rho /R$, and ${l}_{ex}=\sqrt{2A/{\mu }_{0}{M}_{s}^{2}}$ is the exchange length.

We define the skyrmion radius ${R}_{s}<R$ by the equation, $\Theta \left({R}_{s}\right)=\pi /2$, and the reduced radius is ${r}_{s}={R}_{s}/R$. The magnetostatic energy density can be written as a functional^35^

3 $${e}_{m}\left[\Theta \left(r\right)\right]=\frac{1}{\beta }{\int }_{0}^{\infty }dk\left(1-\mathit{exp}\left(-\beta k\right)\right){I}_{z}^{2}\left(k\right)+{\int }_{0}^{\infty }dkkf\left(\beta k\right){I}_{\rho }^{2}\left(k\right)$$

where *β* = *h/R* is the dot aspect ratio, $f\left(x\right)=1-\left(1-\mathit{exp}\left(-x\right)\right)/x$, ${I}_{z}\left(k\right)={\int }_{0}^{1}dr\ r{J}_{0}\left(kr\right){m}_{z}\left(r\right)$, ${I}_{\rho }\left(k\right)={\int }_{0}^{1}dr\ r{J}_{1}\left(kr\right){m}_{\rho }\left(r\right)$, ${J}_{n}\left(x\right)$ are Bessel functions of the first kind, ${m}_{z}\left(r\right)=\mathit{cos}\Theta \left(r\right)$, and ${m}_{\rho }\left(r\right)=\mathit{sin}\Theta \left(r\right)\mathit{cos}\left({\varphi }_{0}\right)$. The first term in Eq. (3) accounts for the magnetic energy of the face charges at the dot top/bottom surfaces. The second term corresponds to extra energy related to the volume and side surface magnetic charges. Only the first term in Eq. (2) contributes to the magnetostatic energy of the vortices, whereas both terms contribute to the energy of the Néel-skyrmions. *I.e.*, the Néel skyrmion magnetostatic energy is always higher than the vortex energy for the same dot magnetic parameters and a finite dot height. Introducing a trial function (skyrmion ansatz) $\Theta \left(\rho \right)$ in the energy functional (2), one can get the energy of the skyrmion configuration as a function of a limited number of the parameters. We use the magnetic skyrmion ansatz corresponding to an exact solution of the 2D exchange Belavin–Polyakov model ^36^.

4 $$\mathit{cos}\Theta \left(r\right)=\frac{{r}_{s}^{2}-{r}^{2}}{{r}_{s}^{2}+{r}^{2}}$$

**Figure 5:**
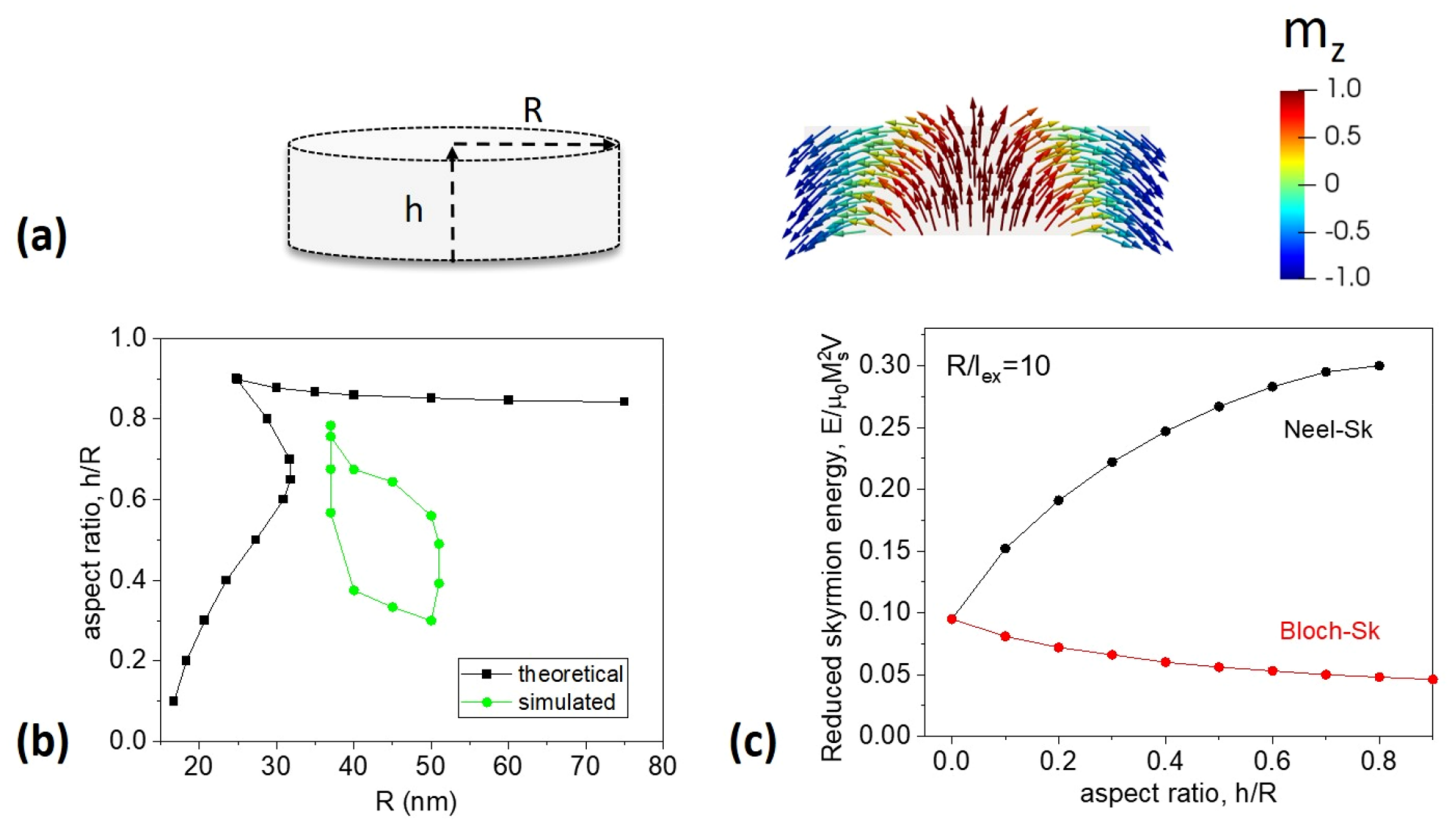
(**a**) Schematic of a cylindrical nanodot and image of the simulated Néel skyrmion magnetization configuration in Permalloy cylindrical nanodot with *R* = 40 nm and *h* = 27 nm. The magnetization is colored according to the value of the *m*_z_ component, as indicated previously. (**b**) State diagram for the Néel skyrmion configuration. Theoretically predicted region is delimited by black curves, while the simulated region—by the green curves. (**c**) The total energy of the Néel skyrmion and vortex state as a function of the dot aspect ratio as predicted by the analytical model.

This ansatz is a good approximation for soft magnetic dots (no magnetic anisotropy) with relatively wide domain walls. In thick magnetic dots one can expect some dependence of magnetization distribution on *z*-coordinate (cf. Fig. 5a) which will diminish validity of the ansatz. However, it allows calculating explicitly the skyrmion energy and equilibrium radius ${r}_{s}$. The calculated energies of the Néel skyrmions and vortex states are presented in Fig. 5a. Both states are metastable because their energies are higher than the energy of the in-plane single domain state $\left[\pi /2\right]=0$. The energy of the Néel skyrmions is always higher than energy of the vortex state due to the magnetostatic energy of the volume magnetic charges.

Therefore, the calculated Néel skyrmion configurations in circular soft magnetic nanodots are metastable or unstable. The area of metastability of the Néel skyrmion in terms of the dot geometrical parameters is presented in Fig. 5b (black squares and line). The Néel skyrmions are high energy metastable states for the large dot radii $R/{l}_{ex}\hspace{0.17em}$> 4–6, and relatively small dot aspect ratio *h/R* < 0.9. The magnetostatic energy increases with *h/R* increasing, eventually making the Néel skyrmion unstable. The exchange energy increases with the dot radius *R* decreasing at fixed *h/R*, making the Néel skyrmion unstable at small *R*.

Further micromagnetic simulations were carried out with the aim to find the geometrical parameters in cylindrical nanodots where a 3D quasi-skyrmion (see Fig. 5a) constitutes an energy minimum state. We were able to find it in a relatively narrow range of geometrical parameters (delimited by the green points and lines in Fig. 5b. These skyrmions are achiral, unlike the case of curved geometry of the dome-shaped dots. Importantly, the metastable Néel skyrmions were obtained in a much smaller region of the dot geometrical parameters than it was predicted by analytical calculations. This may, in the first place, indicate an inaccuracy of the analytical ansatz for thick dots but may be also due to the difficulties of finding initial conditions, which lead to the skyrmion state. See Sect. 5 of “Supplementary Information”.

**Figure 6:**
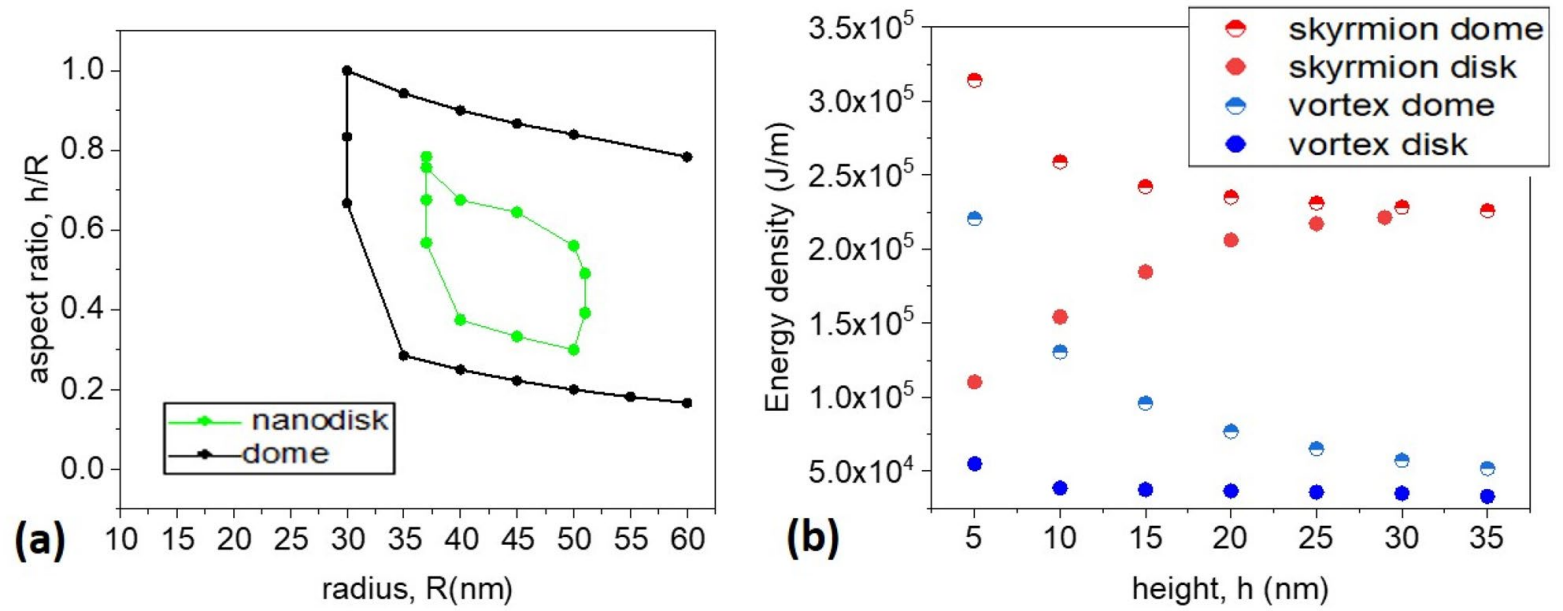
(**a**) Comparison between the regions of existence of Néel skyrmions in dome-shaped and cylindrical dots as a function of the dot radius and aspect ratio parameters (**b**) Energy density of vortex, blue symbols, and skyrmions, red symbols, in dome-shaped (semi-filled symbols) and cylindrical dots (filled symbols). Displayed data correspond to the nanodots of radius *R* = 45 nm.

Finally, we compared dome-shaped and cylindrical dots. Figure 6a presents the comparison between the regions in geometrical parameters space where we found numerically Néel skyrmions in cylindrical and dome-shaped nanodots. The cylindrical dots show a much smaller region of skyrmion existence, demonstrating the importance of the curvature-induced effects in dome-shaped nanodots. Figure 6b compares the energy densities of the vortices and quasi-skyrmions in both geometries, showing smaller energies of both magnetization configurations in cylindrical dots. Additionally, one can notice that while the skyrmion energy density in a cylindrical dot increases as a function of its height in agreement with analytical calculations, Fig. 5c, in dome-shaped dots it decreases due to an additional minimization of the magnetostatic energy.

## Discussion and conclusions

While typically either out-of-plane magnetic anisotropy or Dzyaloshinskii–Moriya interactions are considered to be the necessary ingredients for skyrmion stabilization, confinement effects in magnetic nanostructures can lead to the appearance of topologically non-trivial configurations. Indeed, for 3D structures such as nanowires or nanospheres the occurrence of complex three-dimensional topological magnetization configurations such as Bloch points and 3D vortices has been reported^19–21,23^. In contrast, the 3D Néel skyrmions have not been reported so far in completely soft magnetic structures. The article^20^ reports the formation of skyrmion states in soft hemi-ellipsoidal particles, but at non-zero applied magnetic fields, while Refs. ^26,27^ report them in hemispheres at zero field but with an additional magnetic anisotropy. Here we demonstrate the possibility to observe 3D quasi-skyrmions in both cylindrical and dome-shaped thick nanodots with no need of special magnetic material, i.e., with no DMI and no out-of-plane anisotropy, in particular, in ultra soft Permalloy nanostructures with radius ca. 30–60 nm. The key point is that as the dots are thick, the magnetization configurations are essentially three-dimensional, which allows additional minimization of the magnetostatic energy and emphasizes the curvature effects.

The ground state of these structures is typically either the vortex state or the in-plane single domain state. The quasi-skyrmion is a metastable state at zero field (as most of the skyrmions reported so far). Its projection to the basal plane is a radial vortex. The calculations of 2D topological charge have proved that this structure is topologically non-trivial. The maximum value of 2D topological charge corresponds to dome-shaped nanodots with high aspect ratio, particularly in hemispherical dots it approaches the value 0.9. The region of stability of the quasi-skyrmions in terms of their geometrical parameters is much wider in dome-shaped dots, as compared to cylindrical ones. This underlines the important role of curved surfaces in their stabilization. Additionally, quasi-skyrmions in dome-shaped dots are chiral, while in cylindrical dots they are achiral. Thus, our results bring together novel scientific areas of 3D magnetism^40^ and magnetism in curved geometry^24^. The energy density of Néel skyrmions in cylindrical dots increases as a function of the dot height. A narrow region of stability and high energy make it difficult to observe this magnetization texture experimentally in planar dots. However, skyrmions in dome-shaped nanodots exist in a wide range of geometrical parameters and their energy density decreases as a function of the dot height. Thus, the dome-shaped dots are more promising regarding experimental observations. In relation to this, the possibility to create metastable skyrmions in systems with DMI by magnetic tips has been discussed^37^. A recent experimental study demonstrated the nucleation/stabilization of three-dimensional hedgehog skyrmions in nanodomes by magnetic force microscopy tip^27^. Hence, our work opens novel possibilities for researchers related to the stabilization of non-trivial topological three-dimensional magnetization configurations in completely soft patterned magnetic materials. Without special interactions, especially in curved geometries.

## Methods

We conducted micromagnetic simulations in 3D soft magnetic dots with cylindrical or spherical domed geometry with neither PMA nor DMI. Standard material parameters for Permalloy (Ni_80_Fe_20_) were imposed under no external magnetic field, zero magnetocrystalline anisotropy, exchange stiffness, *A* = 11 pJ/m and saturation magnetization *M*_s_ = 800 kA/m. Most of calculations presented here were carried out with the finite difference code OOMMF^38^ and double-checked by mumax3 program^39^. The geometries of both magnetic elements correspond to the nanodot base radius 10–60 nm and the aspect ratio height/radius ranged from 0.1 to 1, and a 1 nm discretization size. To validate our finite difference-based approach for the studied curved geometry, the effect of the discretization size was studied in Sect. 6 of the “Supplementary Information”. For the simulation of 3D quasi-skyrmions in cylindrical dots, an initial configuration was imposed using the Belavin–Polyakov ansatz: ${\varvec{m}}_{0}=\left(\frac{ 2{r}_{s}r}{{r}_{s}^{2}+{r}^{2}}\widehat{\rho }, 0, \frac{{r}_{s}^{2}- {r}^{2}}{{r}_{s}^{2}+{r}^{2}}\widehat{z}\right)$ taking a skyrmion radius of *r*_s_ = 0.16. For other configurations presented, a configuration close to the desired was imposed as initial configuration and was let evolve to the minimum energy state.

## Supplementary Information

Supplementary Information 1.

Supplementary Information 2.
