# Supplementary material for: 3D quasi-skyrmions in thick cylindrical and dome-shape soft nanodots
Source: Sci Rep. 2022 Mar 2;12:3426. doi: 10.1038/s41598-022-07407-w (PMC8891340; doi:10.1038/s41598-022-07407-w)
Supplement: Supplementary file 1 — Supplementary Information 1. [file 41598_2022_7407_MOESM1_ESM.docx]

# Supplementary information to the manuscript “3D quasi-skyrmions in thick cylindrical and dome-shape soft nanodots.”

# Eider Berganza1,2*, Jose Angel Fernandez-Roldan3,2*, Miriam Jaafar4, Agustina Asenjo2, Konstantin Guslienko5,6 Oksana Chubykalo-Fesenko2

1 Institute of Nanotechnology, KIT, 76344 Eggenstein-Leopoldshafen, Germany

^2^ Instituto de Ciencia de Materiales de Madrid, CSIC, 28049 Madrid, Spain

^3^ Departament of Physics, University of Oviedo, Oviedo 33007, Spain

^4^ Departamento de Física de la Materia Condensada and Condensed Matter Physics Center (IFIMAC), Universidad Autónoma de Madrid, 28049 Madrid, Spain

^5^ [Departamento de](http://www.dfm.ehu.es/) Polímeros y Materiales Avanzados, University of the Basque Country (UPV/EHU), 20018 Donostia, Spain

^6^ IKERBASQUE, the Basque Foundation for Science, 48009 Bilbao, Spain

**Supplementary Information 1**

See the three-dimensional (3D) magnetic configurations of the magnetization in the videos uploaded with the manuscript: i-Skyrmion, ii-flower like state, iii-Bloch Point, and iv-vortex.

**Supplementary Information 2**

The stabilization of the Néel skyrmion-like configuration that satisfies the condition $P\cdot m_{rho}=-1$ has been obtained by the introducing a large out-of-plane uniaxial anisotropy in the base plane of the dome-shaped dot in the micromagnetic model with the value *K*_1_= 10^6^ J/m^3^. Such anisotropy may result from a surface tension due to a mismatch of the lattice parameters when growing dots on some substrate. The initial state was set to a pseudo skyrmion-like configuration with magnetization components in cylindrical coordinates $m_{z}\left( r \right)=\tanh\frac{{r-r}_{o}}{\Delta}$ and $m_{r}\left( r \right)=\mathrm{sech} \frac{{r-r}_{o}}{\Delta}$ and varying the domain wall thickness Δ. The resulting configuration in Fig.S1 (a-c) consists of a radially symmetric Néel skyrmion state with P=-1 and C=+1. The top and bottom view of this state colored by the z-component of magnetization indicate an asymmetric core width of this skyrmion at different heights. Cross section displayed in Fig.S1. (d-e) confirms that the core (in blue) becomes wider at upper cross-section, whereas it keeps a narrower profile at the base of the nanodot.


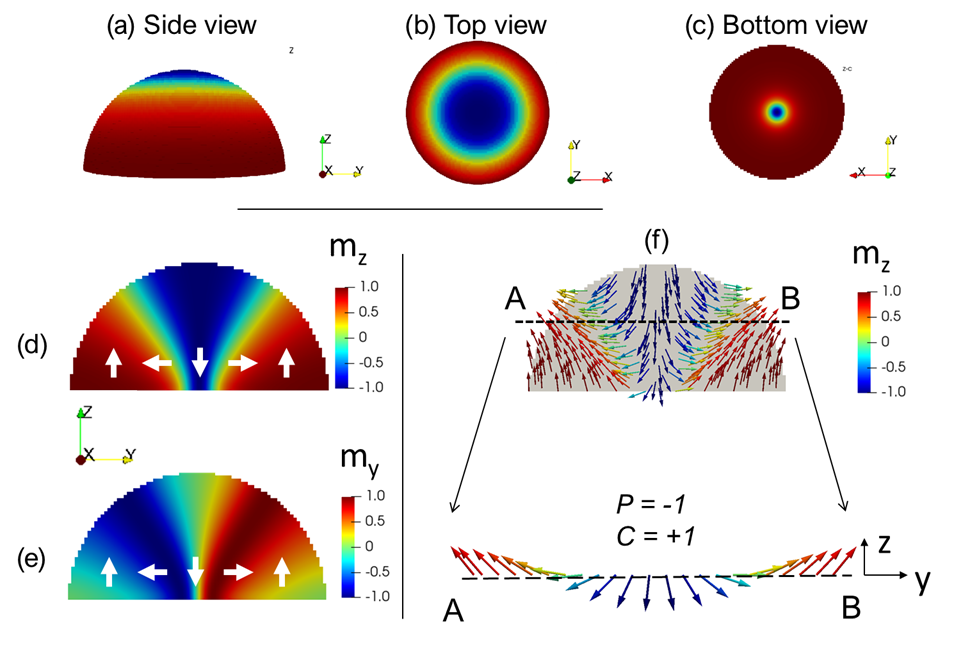


**Fig. S1.** (a) Side, (b) top and (c) bottom views of a hedgehog skyrmion with P=-1 and C=+1 in dome-shaped nanodot with *R*=30 nm *h*=30 nm. The permalloy parameters and strong perpendicular magnetic anisotropy of value 10^6^J/m at the basal plane were used. (d-e) A cross section of this state colored by *z*- and *y*- components of the magnetization. The white arrows schematically indicate the direction of the magnetization. (f) The upper figure shows the magnetization in the cross-section and the bottom figure shows the profile of the skyrmion along the radial direction at a selected height.

A closer inspection of the magnetization on the curved surface of the dome-shaped dot in Fig. S.1.(f) suggests a three-dimensional spin texture dissimilar to typical 3D hedgehog skyrmions and to 3D Néel skyrmion-like configuration in planar nanodots. While this 3D texture is approaching 2D-Néel skyrmions at the basal plane (*z*=0) induced by the planar surface and perpendicular anisotropy, at positive values of the z coordinates the magnetization is mostly normal to the curved surface (particularly at the nanodot top) with the exception of the domain wall region. Importantly, while for planar dots the magnetization distribution and magnetic charges are homogeneous along the out-of-plane coordinate *z*, in dome-shaped nanodots there is a strong dependence on *z* due to the curvature influence. This is illustrated in Fig. S2 below showing magnetization components and magnetic volume charge along radial coordinates at selected cross-sections.

**
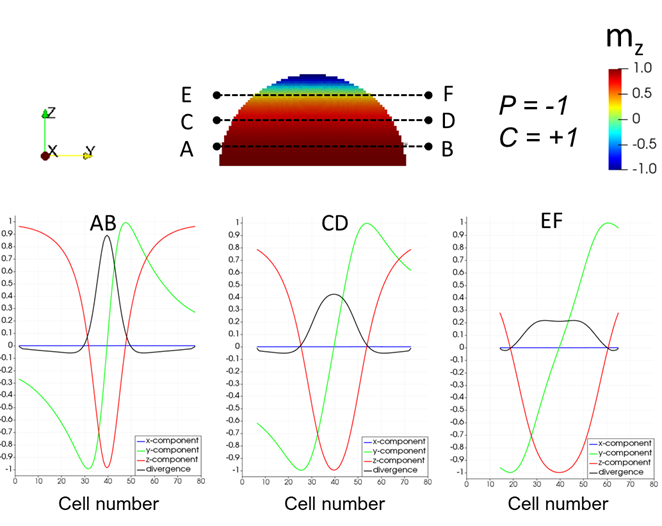
**

**Fig. S2**. Profiles of the volume magnetic charge density (divergence of magnetization) and magnetization components along three selected lines parallel to the *x*-axis AB, CD and EF in the dome-shaped nanodot.

**Supplementary Information 3**

The value of gyrovector is a relevant characteristic since it is related to possible dynamical effects originating from non-trivial topology, for example, to the gyrotropic skyrmion frequency.

The *i* cartesian component of the gyrovector density  $\vec{\gamma}$ (also called vorticity) reads as [S1-3]

$\gamma_{i}=\frac{1}{2}\varepsilon_{ijk} \boldsymbol{m}\cdot(\frac{\partial\boldsymbol{m}}{\partial x_{j}}\times\frac{\partial\boldsymbol{m}}{\partial x_{k}} )$ (S1)

where ***m*** is the direction of the magnetization, $\varepsilon_{ijk}$ the Levi-Civita symbol and the Einstein summation convention is assumed in *j* and *k*. The total gyrovector is the volume integral of (S1) and depends on the system volume. The 2D topological charge *Q* is the integral of the component $\gamma_{z}$ over the cross-section surfaces *z*=const in Eq. (S1).

The increase of the 2D topological charge *Q* in Fig. 4 of the main text is a consequence of the strong gyromagnetic vector density in Fig. S3. The gyromagnetic vector density is mainly aligned along the *z* direction, centered in the nanodot axis and its components increase with the *z*-coordinate increasing. While the 2D topological charge increases with *z* owing to a higher gyrovector density up to a certain maximum, the area of the 2D cross section at each *z* is lower at higher positions and vanishes at the top of the dot and thus the same does the 2D topological charge.


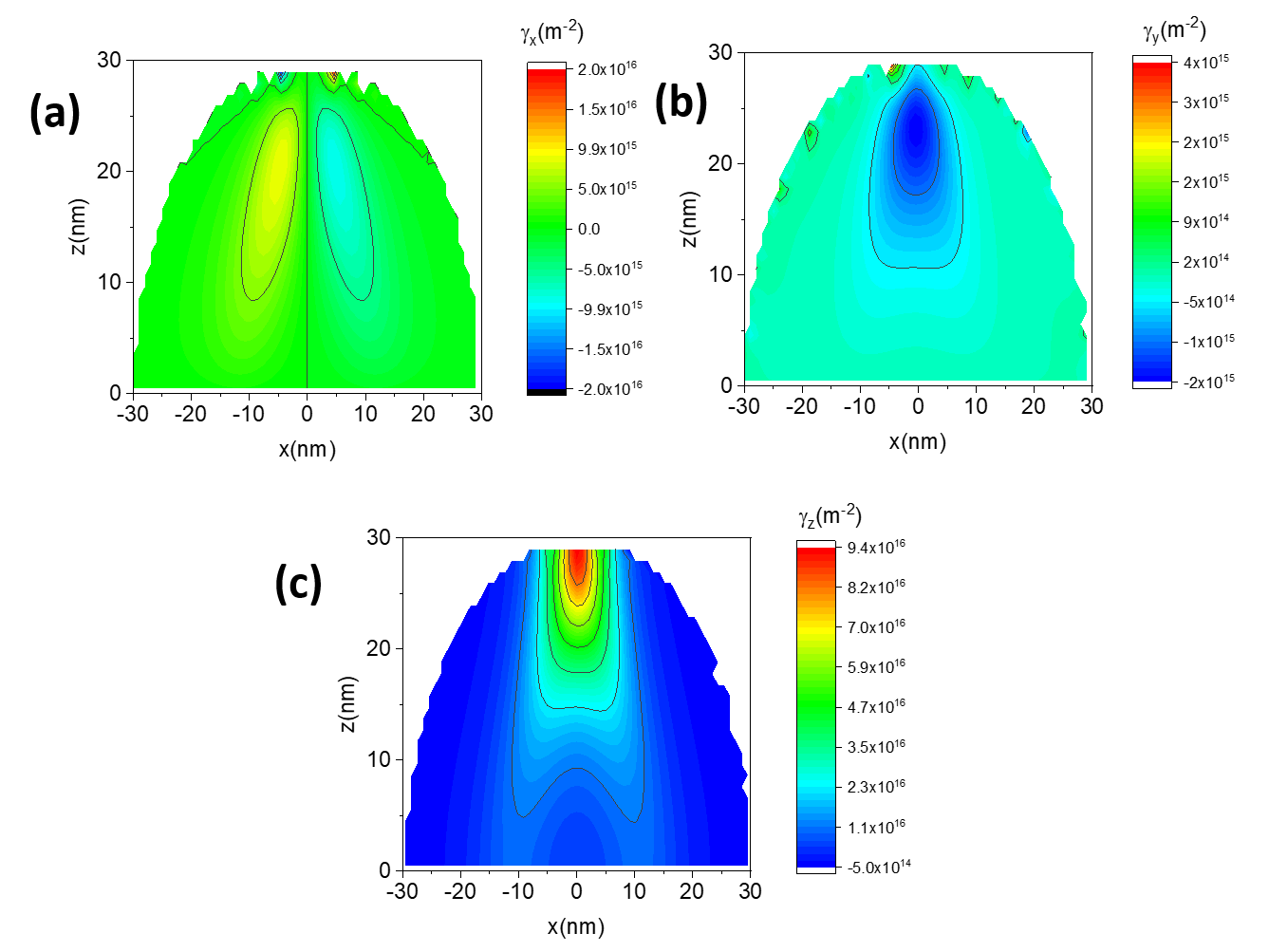


**Fig. S3**. Gyromagnetic field density in a cross section of the dome-shaped nanodot with *R*=50 nm and *h*=40 nm. (a) displays the γ_x_ component, (b) γ_y_ component and (c) γ_z_ component in color scale.

Figure S4 presents a perpendicular gyrovector component as a function of the nanodome diameter. This value depends on the system size. To present a dimensionless value we have normalized the gyrovector to the dot height, showing that the value increases when the height increases.

**
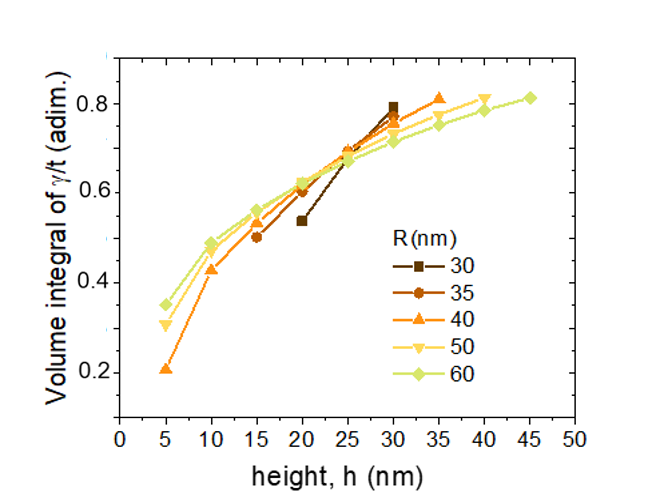
**

**Fig. S4.** Perpendicular component of the gyrovector (normalized to the dot thickness) in nanodots of various diameters.

**Supplementary Information 4**

The 2D topological charges of the 3D vortex states and ‘flower-like’ (out-of-plane) states in dome-shaped nanodots are presented in Fig. S5 (a-b) as a function of the out-of-plane coordinate, *z*. The values confirm a topologically different magnetic configuration from the skyrmion state in Fig. 1a (i). Indeed, the topological charge of the vortex state is maximum at the basal plane reaching a value of 0.5 typical of vortices, and decreases monotonically at higher *z*-values, completely vanishing at the top of the dot. This tendency indicates the 3D vortex magnetic state formed by geometric confinement and the surface curvature, different from vortices in planar nanodots and thin films.

In regard to the flower-like state, we also calculated a small 2D topological charge, mainly on the basal plane and close to it. The low maximum value of 0.2 in the basal plane confirms a different topology than that of the skyrmion. However, while out-of-plane states in thin dots carry a zero topological charge, here the competition between the confinement and magnetostatic energy at both, basal and curved surfaces, induces a 3D magnetic state with non-zero topological charge, and hence, topologically non-trivial. This 2D topological charge decreases with the increase of the z-coordinate and vanishes at the top of the dot, similar to the vortex state. Importantly, notice that the sign of the 2D topological charge in vortices 1,2 and 3, and in flower-like states 1 and 2 changes when the z-component of the magnetization changes.

**
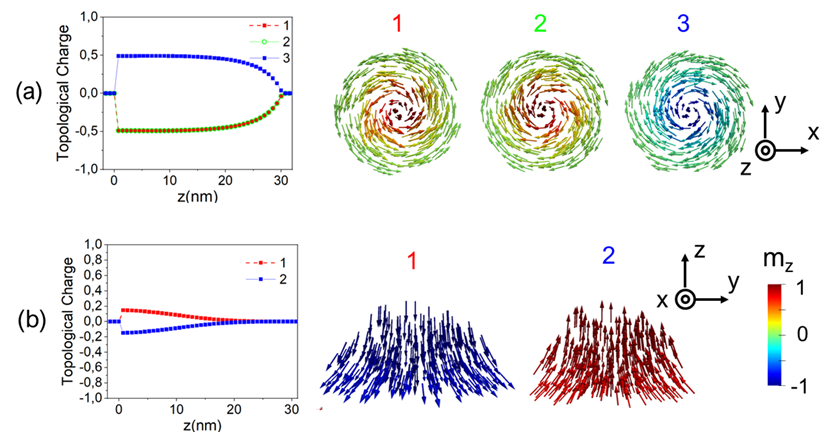
**

**Fig. S5**. 2D Topological charge *Q* of the magnetic vortex states with opposite sense of rotations and opposite polarities (a), and ‘flower-like’ states (b) as a function of the *z*-coordinate in the dot with radius *R*=30 nm and thickness *h*=30 nm.

**Supplementary Information 5**

Computational micromagnetics vs. Belavin –Polyakov ansatz

In order to assess the accuracy of the chosen ansatz for the analytical calculations, in Figure S6 we compare the obtained magnetization configuration components in micromagnetic simulations -along the cylindrical nanodot diameter, for bottom, middle and top sections respectively- to the analytical Belavin-Polyakov model:

$m_{0}=\left( \frac{2r_{s}r}{r_{s}^{2}+r^{2}}\hat{\rho}, 0,\frac{r_{s}^{2}- r^{2}}{r_{s}^{2}+r^{2}}\hat{z} \right)$; **r_s_=R_s_/R=**0.664

As the micromagnetic outcome shows a skyrmion radius dependence as a function of the z coordinate, we can see that in the best fit is obtained in the middle part of the dot. Notice that the computational model reaches more negative values of the axial magnetization than the analytical one, which means that the micromagnetic results are closer to the skyrmion with topological charge 1.

**
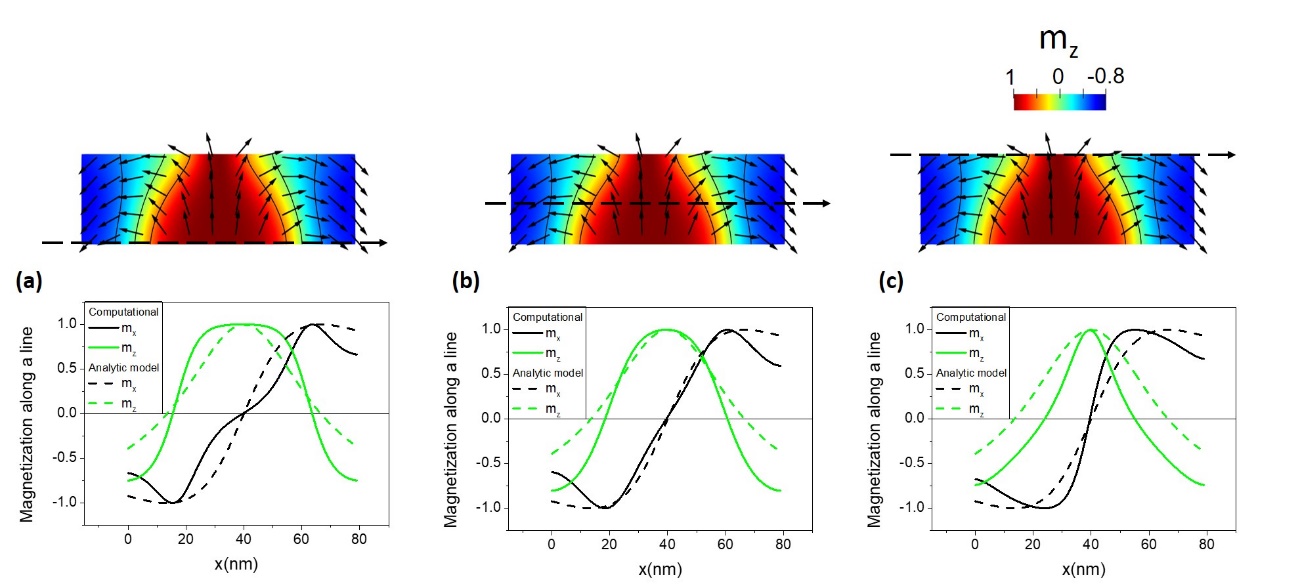
**

**Fig. S6**. Comparison of the magnetization profiles along the diameter in **(a)** bottom, **(b)** middle and **(c)** top sections of the dot, obtained by micromagnetic modeling vs. analytical calculations.

**Supplementary Information 6**

One of the well-known drawbacks of the finite difference simulation approach is the artificial roughness that the model might introduce to simulated curved elements. Thus, we have verified the validity of our results by varying the discretization size. We proved that the magnetic energies for configurations shown in Figure 1 in the main text for a 30 nm diameter hemisphere of the main text converge for a reasonable range of discretization sizes.

Figure S7 shows the calculated energies for each configuration with cell sizes varying between 0.5 and 3 nm. Notice that the in-plane, vortex and flower states are not affected by the chosen discretization (see Fig. S7a, S7b and S7c). However, the Bloch point configuration could not be stabilized for larger discretization values, which yielded a vortex configuration (Fig. S7d). At this point, we would like to point out that the cell sizes larger than 2 nm introduce too much roughness in a small sized (30 nm) hemispherical nanodot.


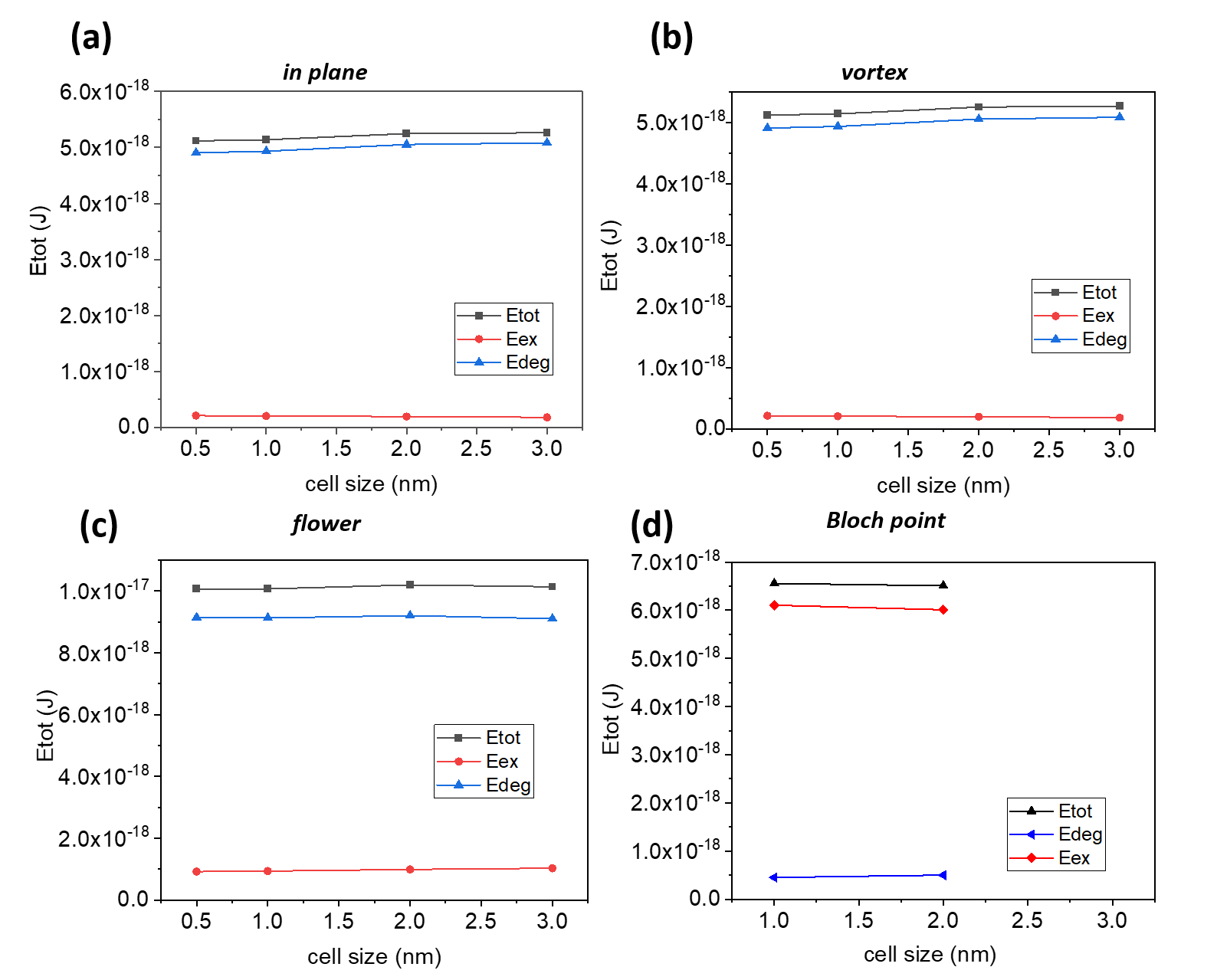


**Fig. S7**. Calculated energy values for different cell sizes, for **(a)** in-plane, **(b)** vortex. **(c)** flower-state and **(d)** Bloch point configurations.

Similarly, in the case of the 3D quasi-skyrmion, the configuration could not be stabilized for bigger cell values. In this regard, a more refined study was conducted in a bigger nanodot (*R*=45 nm and thickness 30 nm) by varying the discretization through smaller steps. As we show in Figure S8, the magnetization configuration was stabilized for a cell range between 0.5 and 1.5 nm size and no remarkable variations were obtained in terms of the energy values.


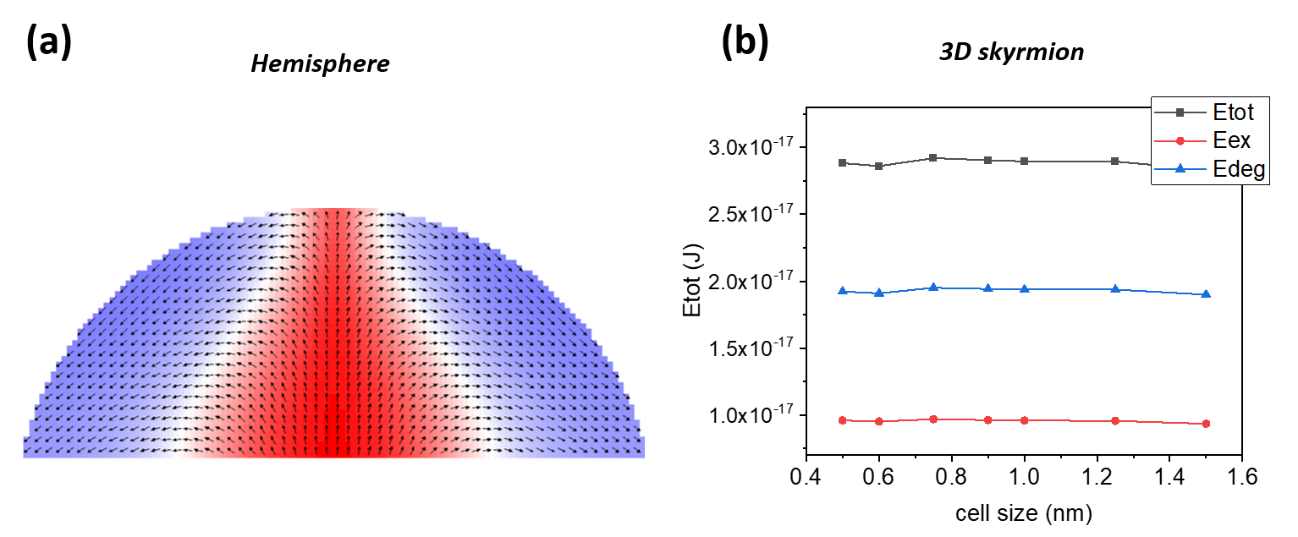


**Fig. S8**. (a) Nanodot of *R*=45 nm and thickness 30 nm showing 3D skyrmion configuration and the induced roughness in the curvature. (b) Calculated total energy for 3D quasi-skyrmion configuration.

**References in SI**

S1. S. Komineas *et al*. Physica D **99**, 81-107 (1996).

S2. B. Göbel *et al*. Phys. Rev. Research **2**, 013315 (2020).

S3. A.M. Kosevich *et al*. Phys. Reports **194**, 117—238 (1990).
